# Supplementary figures and images for: Large-scale genetic characterization of Parkinson’s disease in the African and African admixed populations
Source: medRxiv. 2025 Jan 20:2025.01.14.25320205. Preprint. [Version 2] doi: 10.1101/2025.01.14.25320205 (PMC11759243; doi:10.1101/2025.01.14.25320205)

A

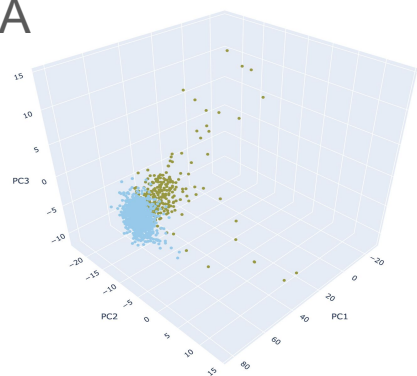

B

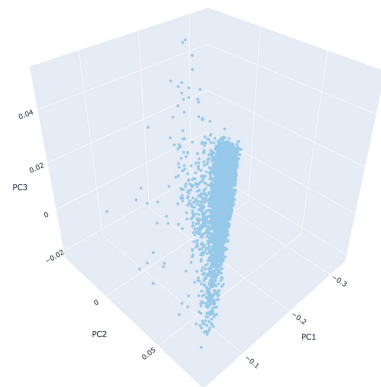

C

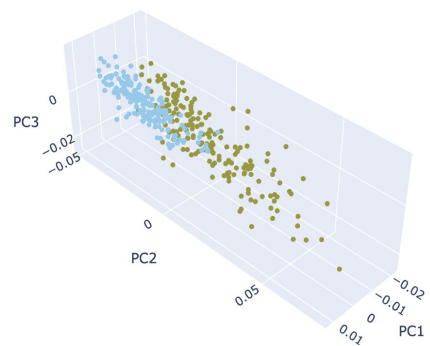

D

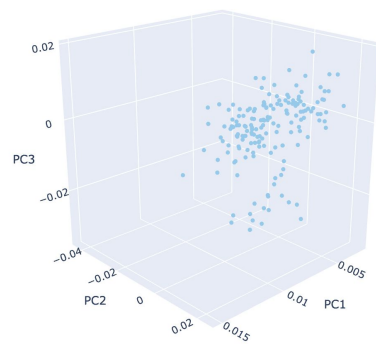

E

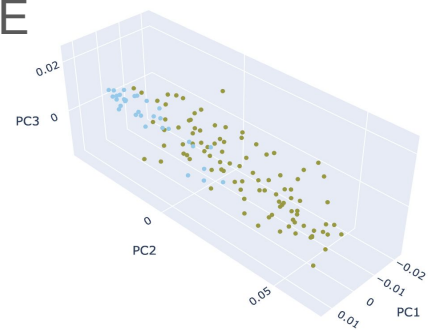

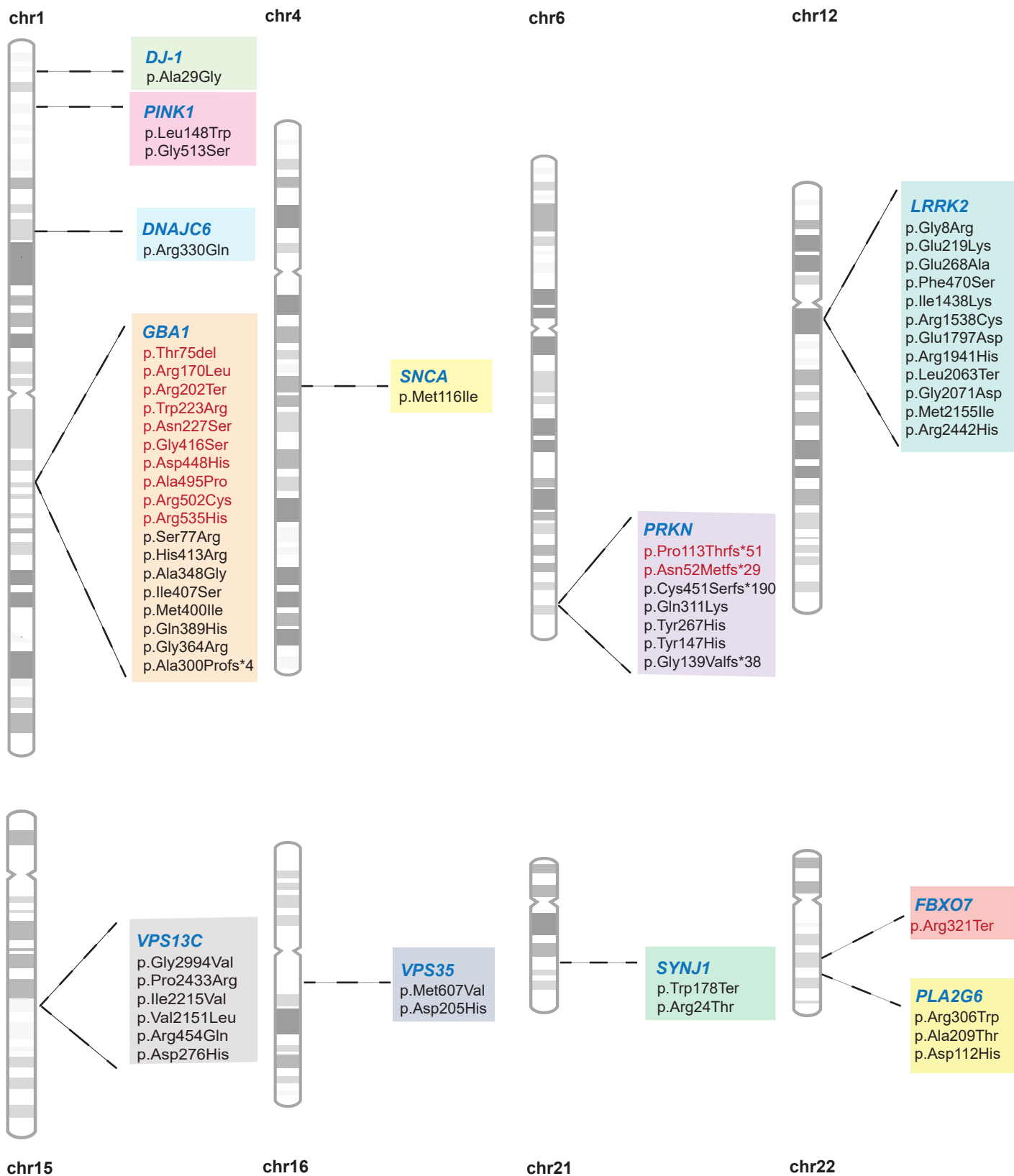

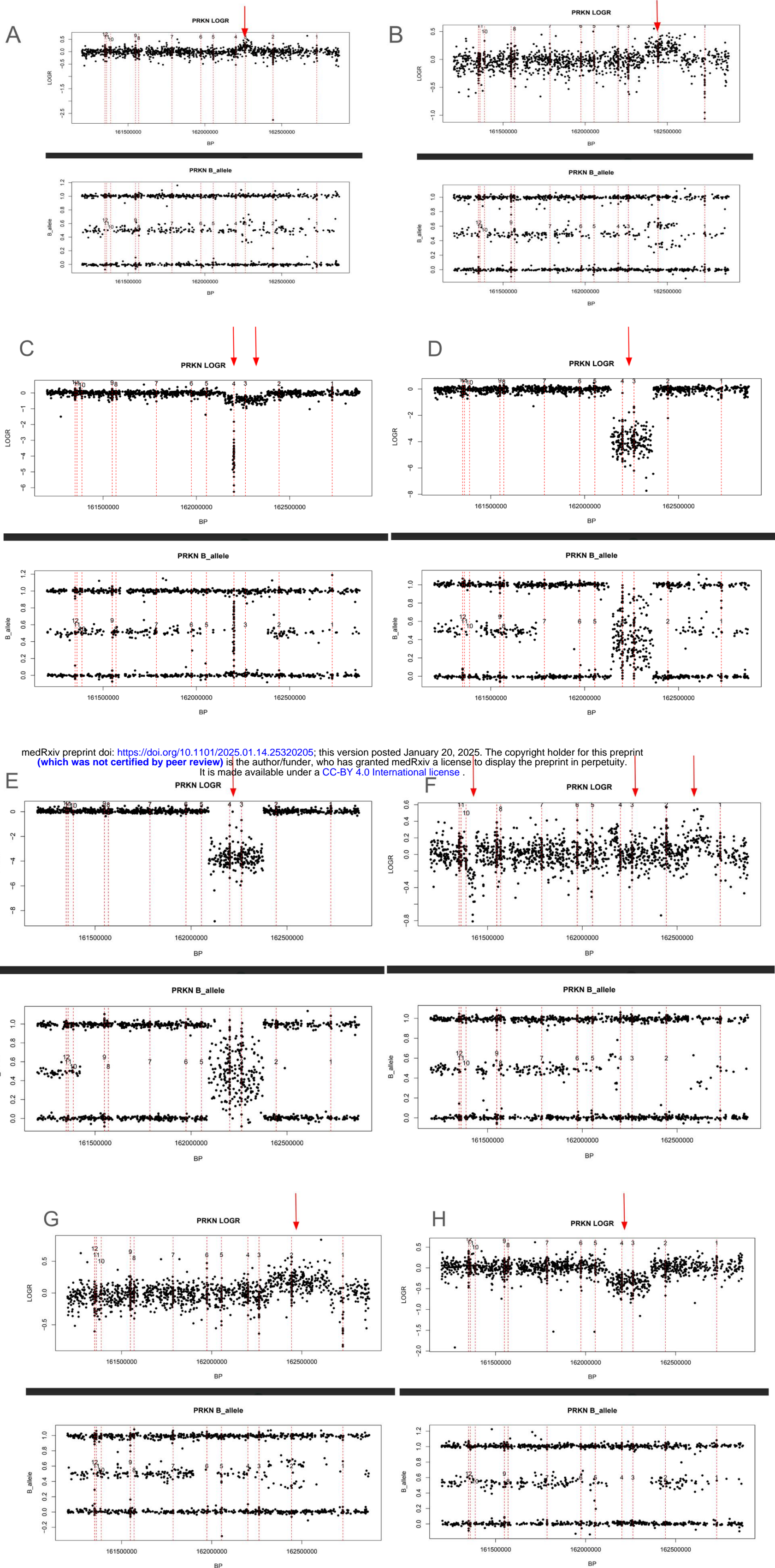

Supplement: Supplement 1 [file NIHPP2025.01.14.25320205v2-supplement-1.pdf]
